# Polysaccharide from *Codium fragile* Induces Anti-Cancer Immunity by Activating Natural Killer Cells

**DOI:** 10.3390/md18120626

**Published:** 2020-12-08

**Authors:** Hae-Bin Park, Juyoung Hwang, Wei Zhang, Seulgi Go, Jihoe Kim, Inho Choi, SangGuan You, Jun-O Jin

**Affiliations:** 1 Shanghai Public Health Clinical Center, Shanghai Medical College, Fudan University, Shanghai 201508, China; haebinpark@yu.ac.kr (H.-B.P.); jyhwang5@yu.ac.kr (J.H.); zhangwei@shphc.org.cn (W.Z.); 2 Department of Medical Biotechnology, Yeungnam University, Gyeongsan 38541, Korea; seulgigo@yu.ac.kr (S.G.); kimjihoe@ynu.ac.kr (J.K.); inhochoi@ynu.ac.kr (I.C.); 3 Research Institute of Cell Culture, Yeungnam University, Gyeongsan 38541, Korea; 4 Department of Marine Food Science and Technology, Gangneung-Wonju National University, Gangneung Daehangno, Gangneung, Gangwon 210-702, Korea

**Keywords:** *Codium fragile* polysaccharide, natural killer cell, cytotoxicity, anti-cancer effect, perforin, granzyme B

## Abstract

Natural polysaccharides exhibit beneficial immune modulatory effects, including immune stimulatory and anti-cancer activities. In this study, we examined the effect of *Codium fragile* polysaccharide (CFP) on natural killer (NK) cell activation, and its effect on tumor-bearing mice. Intravenous CFP treatment of C57BL/6 mice resulted in the upregulation of CD69, which is a marker associated with NK cell activation. In addition, intracellular levels of interferon (IFN)-γ and the cytotoxic mediators perforin and granzyme B were markedly increased in response to the CFP treatment of splenic NK cells. IFN-γ production by NK cells was directly induced by CFP, whereas the upregulation of CD69 and cytotoxic mediators required IL-12. Finally, intraperitoneal treatment with CFP prevented CT-26 (murine carcinoma) tumor cell infiltration in the lungs, without significantly reducing the body weight. In addition, treatment with CFP prevented B16 melanoma cell infiltration in the lung of C57BL/6 mice. Moreover, the anti-tumor effect was diminished by the depletion of NK cells. Therefore, these data suggest that CFP may be used as an NK cell stimulator to produce a phenomenon that contributes to anti-cancer immunity.

## 1. Introduction

Compared to chemically synthesized agents, natural polysaccharides exhibit beneficial activities, such as antibacterial, antiviral, and anti-cancer effects in humans and animals [1,2]. In particular, natural marine polysaccharides, including fucoidan, ascophyllan, and carrageenan, promote immune cell-mediated anti-cancer effects [3,4]. *Codium fragile* is a green alga that is abundantly found in China, Japan, and Korea. This alga contains the following: Polysaccharides (44.1–80.5%); sulfates (3.2–22.2%); proteins (3.0–15.7%); and uronic acids (1.1–4.2%) [5,6]. Sulfated polysaccharides (SP) from *C. fragile* exhibit anti-oxidant, antidiabetic, anti-obesity, and anti-cancer effects in mice [7,8]. In our previous study, fraction 2 of the *C. fragile* polysaccharide (CFP) enhanced the cytotoxic activities of natural killer (NK) cells in vivo [9]. Moreover, CFP also promotes dendritic cell (DC)-mediated antigen-specific immunity in mice, which consequently prevents B16 and CT-26 tumor growth [5]. Although the effects of CFP on NK cell activation have been studied in vitro, the effects of CFP on NK cell activation and NK cell-mediated anti-cancer activities in vivo have not been studied thus far [9].

NK cells are a lymphocyte subtype [10]. Unlike other lymphoid cells, NK cells are involved in innate immunity [11]. They mainly contribute to immunity against viral infections and tumors [12,13]. The stimulation of the Toll-like receptor (TLR) activates NK cells directly or through other immune cells via cytokine-mediated regulation or cell-to-cell interactions [14]. T-cell-derived interferons (IFNs) or DC- and macrophage-derived cytokines are known to promote the activation of NK cells [15,16]. Activated NK cells are characterized by the overexpression of CD69 and secretion of IFN-γ and cytotoxic mediators, such as perforin and granzyme B [17,18]. Cytotoxic mediators released by NK cells kill the target cells; in this process, perforin forms pores in the membrane of target cells, and granzyme B activates apoptosis by cleaving target cell proteins [19,20,21]. Consequently, the activation of NK cells directly promotes the apoptotic killing of target cells [22].

Natural polysaccharides have been shown to activate human and mouse NK cells [23,24]. Our previous studies revealed that CFP induces the activation of mouse DCs in vivo and NK cells in vitro. In this study, we examined whether CFP activates NK cells and enhances anti-cancer immunity in mice.

## 2. Results

### 2.1. CFP Upregulates the Expression of CD69 in Splenic NK Cells

**Figure 1:**
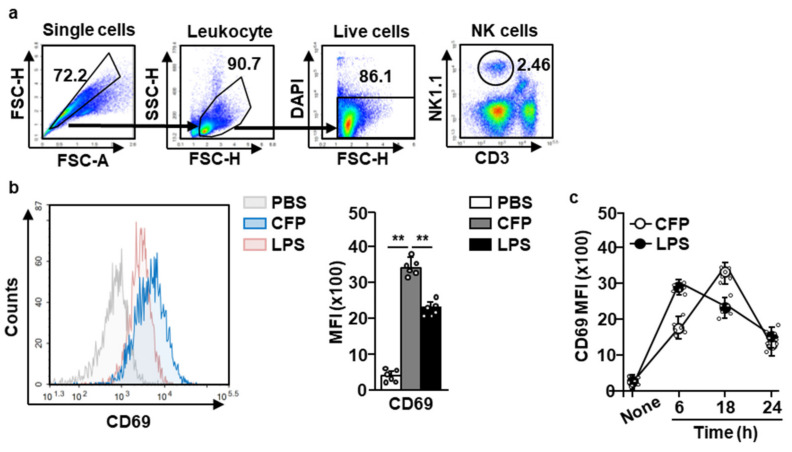
*Codium fragile* polysaccharide (CFP) upregulated CD69 expression in splenic natural killer (NK) cells. C57BL/6 mice were injected intravenously (i.v.) with phosphate-buffered saline (PBS), 50 mg/kg CFP, or 0.1 mg/kg lipopolysaccharide (LPS). (**a**) NK cells in the spleen are defined as NK1.1^+^CD3^-^ leukocytes. (**b**) CD69 expression was measured in splenic NK cells 18 h after CFP and LPS treatments (left panel). The mean fluorescence intensity (MFI) of CD69 is shown (right panel). (**c**) Time-dependent expression of CD69 in splenic NK cells is shown. All data are representative or average values of six independent samples (two mice were used for each of the three experiments, *n* = 6, two-way ANOVA). ** p < 0.01.

To evaluate the effects of CFP on the activation of splenic NK cells, C57BL/6 mice were injected intravenously (i.v.) with phosphate-buffered saline (PBS), 50 mg/kg CFP, or 0.1 mg/kg lipopolysaccharide (LPS). LPS was used as a positive control. Six, eighteen, and twenty-four hours after CFP injection, the spleen was harvested. Splenic NK cells were defined as CD3^−^NK1.1^+^ cells in live leukocytes (Figure 1a). The expression of CD69, which is a specific marker of lymphocyte activation, was found to be significantly increased in splenic NK cells upon CFP treatment (Figure 1b). CD69 expression observed in splenic NK cells upon CFP treatment was highest 18 h after injection, and rapidly reduced 24 h after treatment (Figure 1c). Therefore, these data indicate that CFP can upregulate CD69 expression in splenic NK cells in mice.

### 2.2. CFP Induces the Splenic NK Cell-Mediated Production of Cytotoxic Mediators

**Figure 2:**
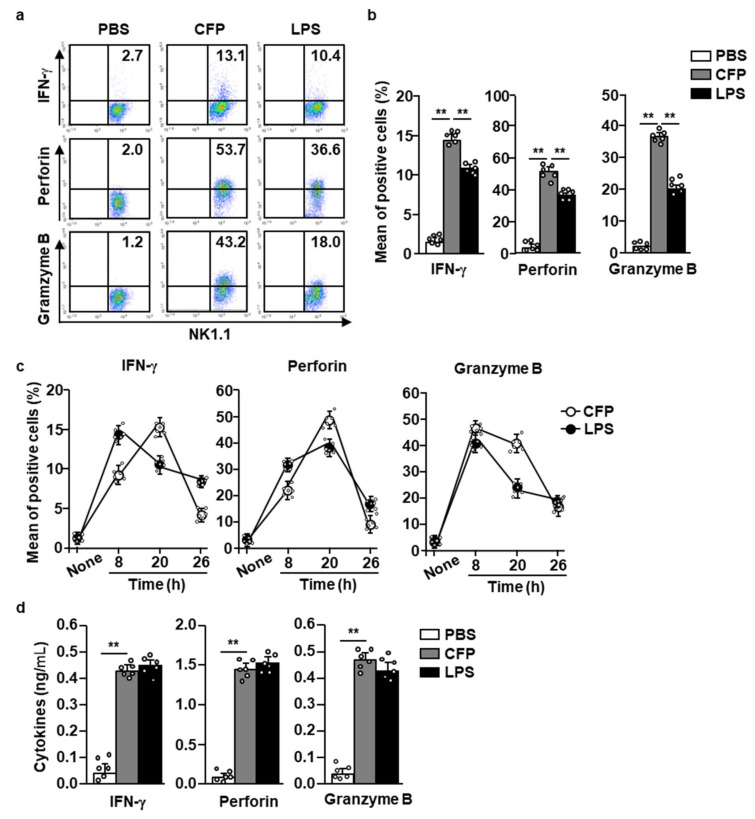
CFP induced the secretion of interferon (IFN)-γ and cytotoxic mediators by NK cells. C57BL/6 mice were injected with PBS, 50 mg/kg CFP, or 0.1 mg/kg LPS (i.v.). Six, eighteen, and twenty-four hours after injection, splenocytes were isolated and then incubated with monensin for 2 h. (**a**) Intracellular levels of IFN-γ, perforin, and granzyme B were analyzed using flow cytometry. (**b**) Mean IFN-γ and cytotoxic mediator-producing NK cells. (**c**) Time-dependent patterns of IFN-γ and cytotoxic mediator-producing NK cells are shown. (**d**) Serum concentrations of IFN-γ, perforin, and granzyme B were measured using an enzyme-linked immunosorbent assay (ELISA). All data are representative or average values from six independent samples (two mice were used for each of the three experiments, *n* = 6, two-way ANOVA). ** *p <* 0.01.

Next, we examined whether CFP can induce the secretion of IFN-γ and other cytotoxic mediators by NK cells. Eighteen hours after CFP injection, the spleen was harvested and splenocytes were incubated with 2 µM monensin for 2 h. The intracellular expression of IFN-γ, perforin, and granzyme B was substantially upregulated upon CFP treatment compared to that in control cells (Figure 2a,b). The percentage of IFN-γ and cytotoxic mediator-producing NK cells increased markedly up to 20 h after CFP injection, but rapidly decreased 26 h after CFP treatment (Figure 2c). Consistent with their intracellular levels, the serum concentrations of IFN-γ, perforin, and granzyme B 18 h were also significantly increased in response to CFP treatment compared to those in PBS-treated controls (Figure 2d). Therefore, these data suggest that CFP can induce the secretion of IFN-γ and cytotoxic mediators by NK cells in mice.

### 2.3. CFP Directly Enhances Splenic NK Cell-Mediated IFN-γ Secretion

**Figure 3:**
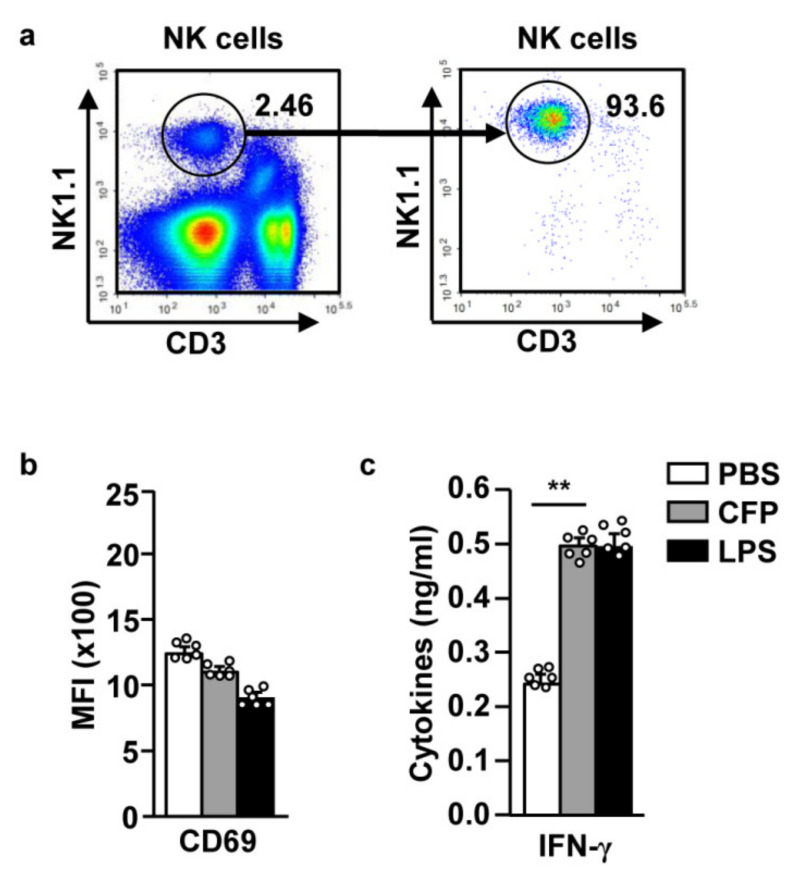
CFP directly induced the secretion of IFN-γ by NK cells. NK cells were isolated from C57BL/6 mice. Isolated NK cells were stimulated with 100 µg/mL CFP and 10 µg/mL LPS for 18 h. (**a**) NK cell purity. (**b**) CD69 expression in NK cells 18 h after CFP treatment. (**c**) IFN-γ concentration in the culture medium. All data are representative or average values of six independent samples (two mice each were used for each of the three experiments, *n* = 6, two-way ANOVA). ** *p <* 0.01.

NK cells are activated by stimulators or cytokines produced by other immune cells. For evaluating the direct effects of CFP on NK cell activation, we isolated splenic NK cells and examined their activation upon CFP treatment. As shown in Figure 3a, splenic NK cells were isolated from splenocytes, and their proportion was found to be >90% of the splenocytes. Isolated NK cells were treated with PBS, 100 µg/mL CFP, or 10 µg/mL LPS for 18 h. CD69 was not upregulated upon CFP treatment (Figure 3b). However, the levels of IFN-γ in the culture medium were significantly increased upon CFP treatment (compared to those observed upon PBS treatment) (Figure 3c). Therefore, these data indicate that CFP directly promotes the secretion of IFN-γ by NK cells; however, the expression of CD69 was indirectly regulated by CFP.

### 2.4. CFP-Induced Activation of NK Cells Is Partially Dependent on IL-12

**Figure 4:**
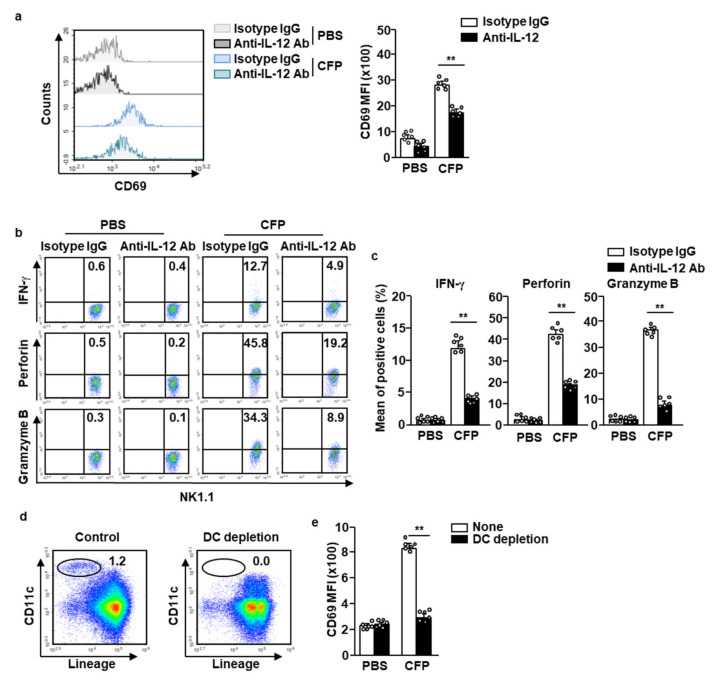
CFP-induced NK cell activation is partially dependent on IL-12. C57BL/6 mice were intraperitoneally (*i.p.)* injected with 5 mg/kg of anti-IL-12p40 antibodies. Two hours after the antibody treatment, the mice received 50 mg/kg of CFP. (**a**) CD69 expression was measured 18 h after CFP treatment by flow cytometry (left panel) and the MFI of CD69 is shown (right panel). (**b**,**c**) The splenocytes were further incubated with monensin for 2 h. (**b**) Intracellular levels of IFN-γ, perforin, and granzyme B were analyzed by flow cytometry. (**c**) Mean percentages of positive cells are shown. (**d**) Dendritic cell (DC) depletion in splenocytes is shown. (**e**) CD69 expression in NK cells was measured by flow cytometry in splenocytes or DC-depleted splenocytes. All data are representative or average values from six independent samples (two mice were used for each of the three experiments, *n* = 6, two-way ANOVA). ** *p <* 0.01.

Because IL-12 is a main contributor to NK cell activation in vivo, we next accessed whether IL-12 is required for CFP-induced NK cell activation. Pretreatment with the anti-IL-12 antibody (Ab) partially inhibited the CFP-induced upregulation of CD69 in splenic NK cells (Figure 4a). Moreover, the intracellular levels of IFN-γ, perforin, and granzyme B were also dramatically decreased upon IL-12 blockade (compared to those observed upon CFP-only treatment) (Figure 4b,c). Since the CD69 expression in NK cells by CFP was partially dependent on IL-12, we next examined the contribution of DC in NK cell activation in the response to CFP. As shown in Figure 4d, the DC was depleted in splenocytes and the cells were further incubated with CFP for 18 h. The upregulated CD69 levels in NK cells by CFP were completely diminished by the depletion of DCs in the splenocytes (Figure 4e). Therefore, these data suggest that DC and IL-12 contribute to the CFP-induced activation of NK cells.

### 2.5. CFP Prevents Tumor Cell Infiltration in Lung Tissues

**Figure 5:**
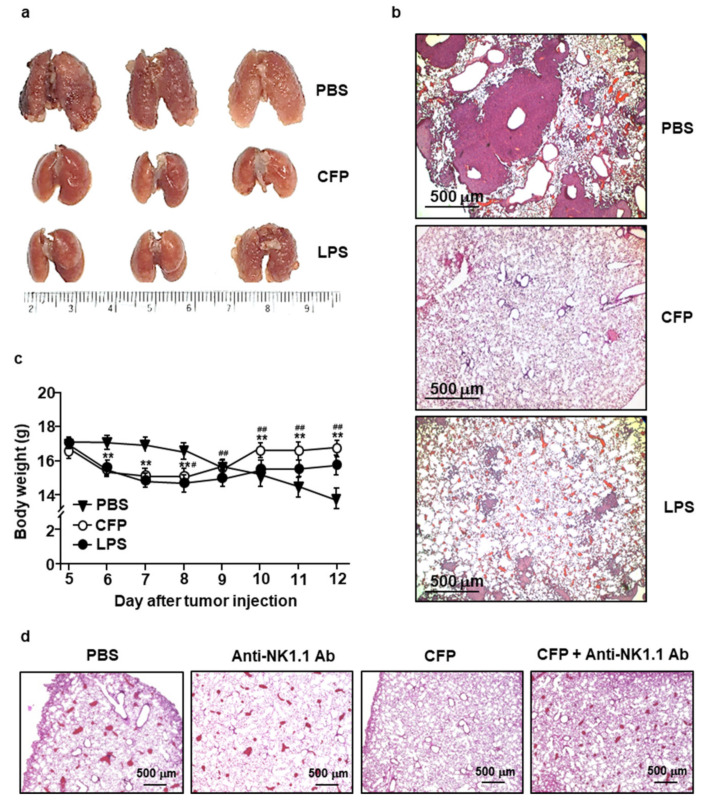
CFP inhibited tumor cell infiltration in lung tissues. BALB/c mice were injected i.v. with 0.3 × 10^6^ CT-26 cells. Five days after the administration of tumor cells, mice were treated intraperitoneally (*i.p.*) with PBS, 50 mg/kg CFP, or 0.1 mg/kg LPS (every day). (**a**) Lung tissues on day 12 after tumor injection. (**b**) Infiltration of CT-26 cells into lung tissues analyzed using H&E staining. (**c**) Changes in body weight measured during treatment with CFP and LPS, respectively. **, *p* < 0.01 (PBS vs. CFP), and ##, *p* < 0.01 (CFP vs. LPS). (**d**) C57BL/6 mice were injected i.v. with 0.5 × 10^6^ B16 cells. Four days after tumor cell injection, 50 µg of anti-NK1.1 Ab was injected at a two day interval. From the 5th day of tumor injection, the mice were treated *i.p.* with PBS and 50 mg/kg of CFP. On day 12 of tumor injection, the lung was harvested and the sections were stained with H&E. All data are representative or average values of six independent samples (two mice were used for each of the three experiments, *n* = 6, two-way ANOVA).

Our results that CFP induced the secretion of IFN-γ and cytotoxic mediators prompted us to examine the anti-cancer effects of CFP. We transplanted 0.3 × 10^6^ CT-26 cells into BALB/c mice via i.v. injection. Five days after the administration of tumor cells, the mice were administered 50 mg/kg of CFP or 0.1 mg/kg of LPS daily. While the lung tissues of PBS-treated mice showed a significant increase in CT-26 tumor masses, the lung tissues of CFP-treated mice did not develop any tumor (Figure 5a). Moreover, a substantially lower infiltration of CT-26 cells into lung tissues was observed in mice that had received CFP treatment compared to that in mice only treated with PBS (Figure 5b). Importantly, the inhibitory effect of CFP on CT-26 tumor cell infiltration in lung tissues was greater than that observed upon LPS treatment (Figure 5a,b). Furthermore, the therapeutic effect of LPS was associated with a decrease in body weight during the treatment of cancer, whereas the same was not true for CFP (Figure 5c). In addition, the CFP treatment in control mice (no tumor) did not induce significant decreases in the body weight compared to PBS-treated control mice (Figure S1).

For an evaluation of the activated NK cell contribution to the anti-cancer effect by CFP in the mice, we depleted NK cells during the treatment of CFP. C57BL/6 mice were injected i.v. with 0.5 × 10^6^ B16 melanoma cells and depleted NK cells by the treatment of anti-NK1.1 Abs (50 µg/100 µL). The depletion of NK cells in anti-NK1.1 antibody-treated mice failed to prevent B16 melanoma cell infiltration; however, treatment with CFP effectively intercepted melanoma cell infiltration into the lungs (Figure 5d). Therefore, these data suggested that the treatment of CFP can inhibit CT-26 and B16 tumor cell growth in the lungs, for which the anti-cancer effect was dependent on NK cell activation.

## 3. Discussions

Polysaccharides extracted from marine organisms have been shown to induce immune stimulatory effects in humans and animals [4,25]. For instance, fucoidan and chitosan are well-studied immune stimulatory marine polysaccharides, and are frequently used as natural healthy foods by humans [26,27]. In this study, we found one more candidate natural polysaccharide—CFP—which promoted the activation of murine NK cells in vivo. In NK cells, CFP upregulated CD69 expression and cytotoxic mediator secretion, which is a phenomenon that inhibited CT-26 tumor cell infiltration in the lungs. More importantly, CFP exhibited a considerably lower cytotoxicity in mice compared to LPS. Therefore, these data demonstrated that CFP can be used as an immune stimulatory molecule in humans and animals.

CFP is a sulfated polysaccharide extracted from green algae, but it does not contain fucose [6,9], which is the dominant sugar in fucoidan—a common sulfated polysaccharide in brown algae [28]. Although CFP and fucoidan contain different monosaccharides and are extracted from different organisms, both exhibit potent immune stimulatory effects. Therefore, CFP can also be used as an immune stimulatory molecule in the treatment of cancer and infectious diseases.

NK cells are innate immune cells [29] which kill pathogens by inducing cytotoxicity and mediate the activation of immune responses, and their use in cytolytic anti-cancer therapy is being investigated because of this ability. NK cell activation is characterized by the upregulation of CD69 and the secretion of cytotoxic mediators, including IFN-γ [30]. Immune stimulatory molecules directly induce NK cell activation; importantly, other immune cells contribute to the activation of NK cells [31]. LPS, Poly I:C, and fucoidan have been shown to promote NK cell activation and IL-12 secretion by DCs, which are well-known stimulatory cytokines for NK cells [32,33,34]. We examined the effect of CFP on NK cell activation using isolated NK cells. Interestingly, differences were observed in NK cell activation in vivo, as well as in vitro; CFP partially promoted the activation of isolated NK cells, whereas CD69 expression was not upregulated and IFN-γ secretion was increased. These incomplete effects of CFP on isolated NK cell activation may be attributed to the absence of other immune cells essential for NK cell activation (which are present in vivo, but are absent in isolated cells). In a previous study, we also found that CFP induces DC activation and IL-12 secretion in mice [5]. IL-12 is known to contribute to NK cell activation [35,36], and we also found that CFP-induced activation of NK cells required IL-12 and DCs. The IL-12 blockade partially inhibited CD69 expression in NK cells, as well as the secretion of IFN-γ and cytotoxic mediators. Moreover, CFP-induced CD69 expression was completely diminished in DC-depleted splenocytes. Therefore, CFP-stimulated DCs and secreted IL-12 may contribute to NK cell activation.

NK cells are cytotoxic immune cells that target cancerous and virus-infected cells [13]. The cytotoxicity of NK cells is mediated via the secretion of cytotoxic mediators, including perforin and granzyme B [21,37]. Perforin released from NK cells via exocytosis creates pores in the membrane of target cells [20], through which granzyme B enters the target cells and leads to their apoptosis via the cleavage of caspases, which are key proteins involved in apoptosis [37,38]. Therefore, NK cell-induced anti-cancer effects are regulated by cytotoxic mediators. CFP substantially increases the secretion of perforin and granzyme B, which may contribute to the observed cytotoxic action against murine CT-26 cells. In future studies, we will further evaluate the anti-cancer effects of CFP-induced perforin and granzyme B by blocking these proteins.

Pattern recognition receptors (PRRs) expressed on immune cells recognize and bind to specific pathogen-associated molecular patterns (PAMPs), thereby resulting in immune cell activation. Complement receptor 3 (CR3) is one of the PRRs expressed on DCs, B cells, T cells, and NK cells [39,40,41]. CFP has been shown to bind to CR3 [9]. Zymosan, which is a fungal surface carbohydrate, is a well-known ligand of CR3. CR3-zymosan binding promotes immune cell activation [42]; however, zymosan is also a ligand for Toll-like receptor 2 (TLR2) [43,44]. As CFP is also carbohydrate, it remains unclear whether CFP can stimulate both CR3 and TLR2. Among all PRRs, TLRs induce immune cell activation more potently upon stimulation with PAMPs. We will further study whether CFP stimulates CR3 and TLR2 for inducing immune cell activation in knock-out mice.

## 4. Materials and Methods

### 4.1. Ethics Statement

The study was carried out according to the guidelines of the Institutional Animal Care and Use Committee at the SPHCC and Yeungnam University Animal Facility, and was approved by the Ethics of Animal Experiments Committee of the Shanghai Public Health Clinical Center (SPHCC). (2018-A049–01) and Yeungnam University (2019–008).

### 4.2. Mice and Cell Lines

C57BL/6 and BALB/c mice were purchased from SPHCC. Some C57BL/6 mice were purchased from Orient Bio (Gyeonggi, Korea). Mice were maintained at a temperature of 22–23 °C under 50–60% humidity and pathogen-free conditions. CT-26 (murine carcinoma cell line; ATCC, CRL-2638) cells were cultured in Roswell Park Memorial Institute (RPMI)-1640 medium supplemented with 100 µg/mL streptomycin, 1 M HEPES, 10% fetal bovine serum (FBS), 2 mM glutamine, and 100 U/mL penicillin at 37 °C in a humidified incubator containing an atmosphere of 5% CO_2_.

### 4.3. Preparation of CFP

Polysaccharides were extracted from *C. fragile* as per protocols described in previous studies [5]. Briefly, *C. fragile* samples were incubated overnight with 90% ethanol at room temperature. After the ethanol was completely evaporated, the samples were extracted with distilled water (65 °C, for 2 h). The water-soluble crude sample was deposited and filtered with ethanol. The precipitated samples were then dissolved in distilled water, and the Sevag method was used for removing free proteins. A DEAE Sepharose fast flow column (17–0709–01, GE Healthcare Bio-Science AB, Uppsala, Sweden) ion-exchange chromatography system was used for sample fractionation. Three fractions (F1, F2, and F3) were separated using chromatography; F2 was found to be the most immunostimulatory polysaccharide fraction and was therefore chosen for use as CFP in further experiments.

### 4.4. Antibodies

Isotype control antibodies (IgG1, IgG2a, or IgG2b), FITC-anti-CD3 (N418), APC-anti-NK1.1 (PK136), APC-Cy7-anti-NK1.1 (53–6.7), APC-Cy7-anti-CD69 (3/23), APC-Cy7-anti-CD49b (16–10A1), PE-Cy7-TCR-β (), APC-Cy7-anti-CD69 (H1.2F3), PE-anti-perforin (GL-1), APC-anti-granzyme B (C18.2), PE-Cy7-anti-IFN-γ (MP5-20F3), and anti-IL-12p40 Abs were procured from BioLegend (San Diego, CA, USA). Anti-NK1.1 Abs were purchased from Invivogen (San Diego, CA, USA).

### 4.5. Flow Cytometric Analysis

Cells were washed with PBS and then pre-incubated with unlabeled isotype control antibodies and Fc-blocked antibodies for 15 min, followed by probing with fluorescence-conjugated antibodies on ice for 30 min. After washing with PBS, the cells were treated with 4′,6-diamidino-2-phenylindole (DAPI) (Sigma-Aldrich, St. Louis, Missouri, USA) and analyzed on FACS Aria II (Becton Dickinson, Franklin Lakes, NJ, USA) and NovoCyte (ACEA Biosciences Inc., San Diego, CA, USA) flow cytometers.

### 4.6. Splenic NK Cell Analysis

Spleen tissue was ground between two coverslip glasses, and cells extracted from the tissue were transferred into 10 mL red blood cell (RBC) lysis buffer (Invitrogen, San Diego, CA, USA) after filtering through a nylon mesh (100 nm pore size). The pellets were resuspended in 5 mL RBC lysis buffer to remove any remaining RBCs. Then, the cells were centrifuged at 1800× *g* for 10 min to obtain splenocytes. These were then probed with anti-CD3 and -NK1.1 monoclonal antibodies (mAbs). Spleenic NK cells were defined as NK1.1^+^CD3^−^ cells.

### 4.7. Intracellular Cytokine Analysis

Splenocytes harvested from C57BL/6, BALB/c mice were incubated with golgistop (2 µM of monensin, BioLegend, San Diego, CA, USA) for 2 h at 37 °C. Washed pellets were stained using the Zombie Violet Fixable Viability Kit (BioLegend, San Diego, CA, USA) at room temperature for 15 min to identify dead cells. Pellets stained with Zombie were washed and stained with surface antibodies for 20 min at 4 °C. After washing with PBS, the cells were fixed and permeabilized using Cytofix/Cytoperm buffer (eBioscience, San Diego, CA, USA) for 30 min. The cells were washed with Perm/Wash buffer (eBioscience, San Diego, CA, USA), and incubated with intracellular antibodies, PE-Cy7-anti-IFN-γ, PE-anti-perforin, and APC-anti-granzyme B at room temperature for 30 min. After washing with Perm/Wash buffer, the cells were resuspended in PBS and analyzed by flow cytometry. This experiment was performed after pre-staining the cells with unconjugated isotype control antibodies and Fc receptor binding antibodies to block the nonspecific binding of fluorescence-conjugated antibodies.

### 4.8. Isolation of NK Cells

NK cells were purified from splenocytes using a FACS Aria II cell sorter (Becton Dickinson, San Diego, CA, USA). Briefly, NK cells were stained with FITC-conjugated anti-NK1.1 and -CD3 antibodies. NK1.1^+^CD3^−^ cells were isolated, and NK cell preparations revealed >90% purity.

### 4.9. ELISA

Heart blood of the mice was harvested 6 or 18 h after the treatment of CFP and sera were harvested after centrifugation (2000× *g* for 10 min). Concentrations of IFN-γ in serum and culture media were analyzed in triplicate using standard ELISA kits (Biolegend, San Diego, CA, USA). The perforin ELISA kit was purchased from Abbkine (Wuhan, China). The granzyme B ELISA kit was obtained from LSBIO (Seoul, Korea).

### 4.10. Tumor Treatment

BALB/c were injected i.v. with 0.3 × 10^6^/100 mL CT-26 carcinoma cells. From the 5th day onwards, the mice were intraperitoneally treated with PBS, 50 mg/kg CFP, or 0.1 mg/kg LPS, and monitored until the 12th day of treatment. The mice were then euthanized and their lungs were harvested for further analysis. For evaluating the contribution of NK cells in the anti-cancer effect by CFP treatment, C57BL/6 mice were intravenously injected with 0.5 × 10^6^/100 mL B16 melanoma cells. From the 4th day of tumor injection, the mice were treated *i.p.* with 50 µg/100 µL of anti-NK.1.1 Ab at a two day interval. They were regularly treated with PBS or 50 mg/kg CFP on day 5 of tumor injection. After the 12th day of tumor injection, the lungs were harvested for further analysis.

### 4.11. Statistical Analysis

All data are expressed as means ± the standard error of the mean (SEM). One- or two-way analysis of variance (ANOVA), followed by Tukey’s multiple comparison test and a Mann–Whitney *U*-test, were used for the analysis of datasets by SPSS (IBM, Armonk, NY, USA). *p* < 0.05 was considered significant.

## 5. Conclusions

In conclusion, in this study, we found that CFP activated NK cells, and this phenomenon included the upregulation of CD69 expression and production of cytotoxic mediators, which was dependent on DC and IL-12. Moreover, treatment with CFP suppressed CT-26 and B16 tumor cell infiltration in lung tissues in BALB/c and C57BL/6 mice, respectively. Therefore, these data demonstrated that CFP may serve as an immune stimulatory molecule that enables cancer treatment through NK cell activation.

## Supplementary Materials

The following are available online at https://www.mdpi.com/1660-3397/18/12/626/s1, Figure S1: Changes of body weight by PBS, CFP and LPS treatment in the mice.
