# Supplementary material for: Polysaccharide from Codium fragile Induces Anti-Cancer Immunity by Activating Natural Killer Cells
Source: Mar Drugs. 2020 Dec 8;18(12):626. doi: 10.3390/md18120626 (PMC7763488; doi:10.3390/md18120626)
Supplement: Supplementary file 1 [file marinedrugs-18-00626-s001.pdf]

marine drugs

Supplementary figure

Polysaccharide from *Codium fragile* Induces Anti-Cancer Immunity by Activating Natural Killer Cells

Hae-Bin Park [1,2,3], Juyoung Hwang [1,2,3], Wei Zhang [1], Seulgi Go [2,3], Jihoe Kim [2,3], Inho Choi [2,3], SangGuan You [4,*] and Jun-O Jin [1,2,3,*]

[1] Shanghai Public Health Clinical Center, Shanghai Medical College, Fudan University, Shanghai 201508, China; haebinpark@yu.ac.kr (H.-B.P.); jyhwang5@yu.ac.kr (J.H.); zhangwei@shphc.org.cn (W.Z.)
[2] Department of Medical Biotechnology, Yeungnam University, Gyeongsan 38541, Korea; seulgigo@yu.ac.kr (S.G.); kimjihoe@ynu.ac.kr (J.K.); inhochoi@ynu.ac.kr (I.C.)
[3] Research Institute of Cell Culture, Yeungnam University, Gyeongsan 38541, Korea
[4] Department of Marine Food Science and Technology, Gangneung-Wonju National University, Gangneung Daehangno, Gangneung, Gangwon 210-702, South Korea
* Correspondence: umyousg@gwnu.ac.kr (S.Y.); jinjo@yu.ac.kr (J.-O.J.); Tel.: +82-33-640-2853 (S.Y.); +82-53-810-3033 (J.-O.J.); Fax: +82-33-640-4769 (S.Y.); +82-53-810-4769 (J.-O.J.)

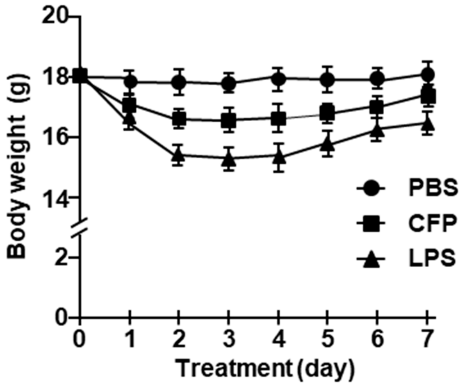

Figure S1. Changes of body weight by PBS, CFP and LPS treatment in the mice. BALB/c mice were injected *i.p.* with PBS, 50 mg/kg CFP, or 0.1 mg/kg LPS (every day). Changes in body weight measured during treatment with PBS, CFP and LPS.
